# Endocrine Regulation in the Ovary by MicroRNA during the Estrous Cycle

**DOI:** 10.3389/fendo.2017.00378

**Published:** 2018-01-22

**Authors:** Derek Toms, Bo Pan, Julang Li

**Affiliations:** ^1^Faculty of Veterinary Medicine, Department of Comparative Biology and Experimental Medicine, University of Calgary, Calgary, AB, Canada; ^2^Department of Animal Biosciences, University of Guelph, Guelph, ON, Canada; ^3^College of Life Science and Engineering, Foshan University, Foshan, China

**Keywords:** ovarian, microRNA, posttranscriptional, steroidogenesis, gonadotropin, granulosa cell, ovulation

## Abstract

Hormonal control of the estrous cycle that occurs in therian mammals is essential for the production of a functional egg. Supporting somatic cell types found within the ovary, such as granulosa and theca cells, respond to endocrine signals to support oocyte maturation and ovulation. Following the release of the egg, now available for fertilization, coordinated hormonal signaling between the mother and putative embryo are required for the establishment of pregnancy. If no conception occurs, both the ovary and uterus are “reset” in preparation for another cycle. The complex molecular changes that occur within cells in response to hormone signaling include a network of non-coding microRNAs (miRNAs) that posttranscriptionally regulate gene expression. They are thus able to fine-tune cellular responses to hormones and confer robustness in gene regulation. In this review, we outline the important roles established for miRNAs in regulating female reproductive hormone signaling during estrus, with a particular focus on signaling pathways in the ovary. Understanding this miRNA network can provide important insights to improving assisted reproductive technologies and may be useful in the diagnosis of female reproductive disorders.

## Introduction

The ovary’s role in producing a fertilizable egg and in signaling to prepare for pregnancy involves complex cascades of hormonal regulation. Germ cells direct the formation of embryonic ovaries, and during this time, each primordial oocyte becomes surrounded by a layer of granulosa cells that will support its development. Cells of the follicle, the functional units of the ovary, proliferate and differentiate largely autonomously throughout early life [reviewed in Ref. (1)].

Studies in a number of species have demonstrated that regulation of ovary formation and early growth is driven by cellular microRNAs (miRNAs) [reviewed in Ref. (2)]. Naturally occurring small molecules ~22 nucleotides in length, miRNAs are involved in almost all developmental and pathological processes in animals by regulating the majority of animal mRNA (3, 4). The biogenesis and function of these small molecules has been described at length and the reader is referred to two excellent reviews on the subject (5, 6). As an RNA molecule, miRNAs are amenable to a number of detection methods, including northern blot, *in situ* hybridization, and reverse-transcription PCR. These, along with high throughput methods like microarray hybridization and RNA sequencing, have been used to detect these small RNA molecules at all stages of ovarian development in a number of important model organisms and livestock (2, 7–11).

At puberty, increasing levels of follicle-stimulating hormone (FSH) released by the pituitary, act on the FSH receptor (FSHR) to integrate various signaling pathways driving follicle growth. Crosstalk between granulosa cells and the oocyte drives its development to the first mitotic stage of meiosis, also known as the germinal vesicle (GV) stage. Granulosa cells ramp up their production of estrogens, specifically the essential female reproductive hormone estradiol (E2) (12, 13). Androgens produced by ovarian theca cells, an important structural and steroidogenic cell type, are converted into E2 in granulosa cells *via* aromatization by cytochrome P450 aromatase (aromatase; coded by the *CYP19A1* gene) (14). As an endocrine hormone, E2 modulates the structure and function of female reproductive tissues, such as the ovary, uterus, and oviduct. It is also one of the principle determinants influencing the function of pituitary neurons, enabling these cells to exhibit fluctuating patterns of biosynthetic and secretory activity [reviewed in Ref. (15)]. Endocrine regulation of ovarian function involving FSH and E2 define the follicular phase.

Increasing E2 serum level induces release of a surge of luteinizing hormone (LH) from the pituitary that acts on dominant follicles and initiates ovulation. The LH receptor (LHR; coded by the *LHCGR* gene) is expressed in granulosa and theca cells of antral follicles based on interactions between FSH and insulin-like growth factor (IGF)-1 signaling [reviewed in Ref. (16)]. Maturation of the oocyte involves resumption of its meiotic arrest until the second meiotic metaphase (MII) where it will again pause. At this time, the oocyte and the layers of closely associated cumulus granulosa cells are released as a cumulus-oocyte complex (COC) from the ruptured follicle and the remaining mural granulosa cells and theca cells begin to differentiate toward their ultimate luteal fate.

This important developmental phase, and the impact these events have on fertility have prompted many investigators to probe the role miRNA plays during periovulatory oocyte development and ovulation. Ovary-specific knockout of DICER resulted in dysfunctional folliculogenesis, oocyte maturation, and ovulation, and thus infertility (17–19). Targeted deletion of the canonical miRNA processing protein DGCR8, however, had no significant adverse effects on mouse oocyte maturation and mRNA levels were unchanged. Although litter size was reduced in mutant animals, DGCR8-deficient oocytes could be fertilized and produced healthy offspring (20). In contrast to oocytes lacking DICER, which processes other small RNA species besides miRNA, it seems apparent that the role of miRNA in the oocyte is limited. Growing evidence supports the notion that the influence of miRNA on ovarian function primarily occurs through their actions on ovarian somatic cells such as granulosa cells, and these cells may be the source of many oocyte miRNAs (21).

The ruptured follicle is remodeled into a corpus luteum (CL) that now produces large amount of progesterone (P4) to prepare the uterine endometrium for the conceptus. Progesterone also acts on somatic cells of the ovary by interacting with numerous receptors, the most well characterized of which is the nuclear progesterone receptor (PGR). Both FSH and LH upregulate the expression of PGR, which itself regulates a cascade of genes associated with vascular development, ovulation, and metabolism [reviewed in Ref. (22)]. The CL will ultimately degenerate *via* granulosa apoptosis, either during pregnancy when the bulk of maternal hormone production shifts to the placenta, or more quickly as the estrous cycle resumes [reviewed in Ref. (23, 24)]. High levels of P4 characterize the luteal phase and are essential for establishing pregnancy.

While the role of miRNA is minimal in the oocyte [reviewed in Ref. (25)], it has become increasingly evident that the somatic components of the ovary have a complex network of miRNAs that fine-tune cell behavior (26). At each stage of ovary development and during the estrus cycle, dynamic cellular responses to endocrine and paracrine signaling now appears to include a prominent role for miRNA. Of particular interest here are those miRNA involved in regulating the complexities of hormonal signaling necessary for follicle growth, ovulation, and the establishment of pregnancy. Because of the big impact these small molecules may have on fertility as well as physiological processes, numerous investigators have begun interrogating miRNA specifically in the context of the hormonal ovary. In this review, we will highlight many of the studies that have advanced our understanding of this interconnected miRNA–endocrine signaling network in the somatic cells of the ovary.

## Regulation of Ovarian Cycling by miRNA

### High Throughput Screens, Differential Expression

Early work to identify important miRNA players in follicle growth and ovulation took a global approach and profiled miRNA expression at important stages of the estrus cycle. Work done in rodents and livestock identified hundreds of differentially expressed miRNAs following stimulation with FSH, hCG, and during natural and induced ovarian cycling (27–30). Granulosa cells collected immediately before and 4 h after an ovulatory surge of LH/hCG exhibit differential miRNA gene expression patterns (31), suggesting a role in ovulation. Using a large-scale platform approach, it was shown that 51 miRNAs have suppressive effects on estradiol biosynthesis in cultured human granulosa cells (32). Of 80 miRNAs transfected into granulosa cells, 36 significantly inhibited progesterone release while 10 miRNA showed inductive effects. This trend held up for testosterone where over 60% of miRNAs attenuated hormone release. Only miR-107 increased culture levels of testosterone while no miRNA led to increased estradiol secretion (32). Such screening experiments reveal changing miRNA networks that are related to both hormone production and response in ovarian somatic cells.

**Figure 1:**
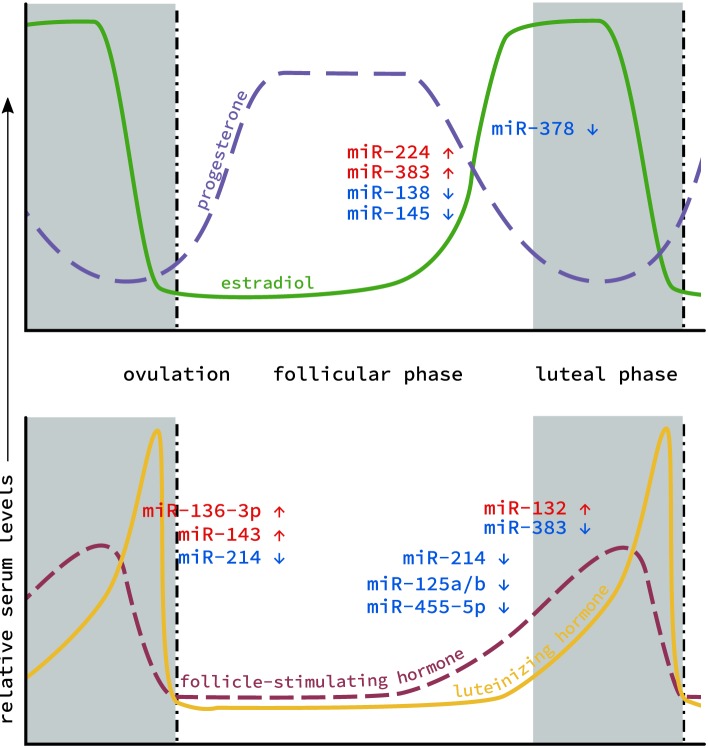
MicroRNA (miRNA) levels in the ovary during the estrous cycle. Levels of the two main classes of endocrine hormones, steroid (top) and gonadotropin (bottom), fluctuate throughout the course of the cycle. Similarly, miRNA expression has also been shown to fluctuate between the follicular phase (gray), through ovulation (dotted line) to the luteal phase (white). Individual miRNAs are depicted based on whether their expression increases (red) or decreases (blue) during this cycle.

Because of their ease of detection, particularly compared to hormone levels in small samples (e.g., spent media from embryo culture) or in associated somatic cells that are routinely discarded (i.e., granulosa cells), interest has grown in using miRNA as a non-invasive biomarker of oocyte quality. Characterization of miRNA profiles have been conducted on patients undergoing oocyte retrieval for various reproductive technologies (33). miRNAs associated with oocyte development or serum hormone levels also insinuate various miRNAs as playing important roles in ovarian function. These studies established links between hormone signaling and miRNA expression (Figure 1), and laid groundwork for the interrogation of specific miRNAs and the roles they might be playing in the ovary.

### Follicular Phase

**Figure 2:**
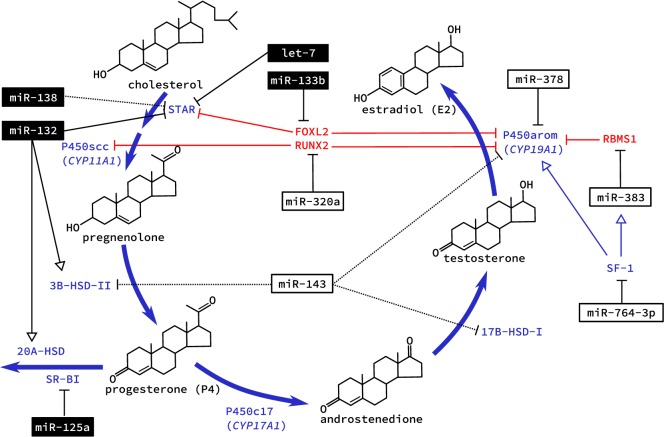
Regulation of steroidogenesis in the ovary by microRNA (miRNA). The two main steroid hormones synthesized in the ovary are estradiol and progesterone. Biosynthesis of these two hormones is regulated in the ovary by miRNA at all levels of regulation including transcription of genes coding for essential enzymes, direct posttranscriptional regulation of these enzymes, and expression of enzymes that catalyze the conversion of these hormones to inactive metabolites. miRNAs that are known to be regulated by cyclic adenosine monophosphate are shown in black boxes; dashed lines indicated an observed effect with unknown mechanism. Note that several metabolic intermediates (e.g., estrone, dehydroepiandrosterone) have been omitted for clarity.

With the increase in bioavailable FSH after puberty, granulosa cells are targeted specifically as they alone express the FSHR. This G-protein coupled receptor integrates various signaling pathways by increasing intracellular cyclic adenosine monophosphate (cAMP) [reviewed in Ref. (34)]. Hormone production also occurs in the adrenal glands, which share many of the same features and pathways as the ovary (35, 36). This was the basis of several studies in primary adrenal miRNA regulation, although key findings were confirmed in primary rat granulosa cells (37, 38). Addition of cAMP to cultured cells was studied for its effect on miRNA expression. The first study identified miR-125a-5p, miR-125b, miR-455-5p, and miR-145, as being down regulated in granulosa cells after exposure to cAMP (37). Similarly, miR-138 was also later confirmed to decrease with administration of cAMP *in vitro* and predicted to target the message for steroidogenic acute regulatory protein (STAR) (38). STAR is essential for transporting cholesterol to the inner mitochondrial membrane where it can be cleaved by the cholesterol side-chain cleavage enzyme (coded by the *CYP11A1* gene), and was recently shown to be regulated by interactions between the miRNA let-7 and the non-coding miRNA “sponge” H19 in a human granulosa cell line (39). Validated targets of cAMP-induced miRNAs in rat granulosa cells were sterol regulatory element-binding protein (SREBP)-1c, involved in regulating *de novo* lipogenesis, and low density lipoprotein receptor (LDLR) that regulates the delivery of cholesterol to cells. However, of two miRNAs shown to target these genes in adrenal cells, miR-132 and miR-214, respectively, only miR-214 showed decreased expression with cAMP treatment in granulosa cells along with a corresponding increase in SREBP-1c and LDLR protein levels (38). While additional ovarian-specific functions of miR-214 have yet to be elucidated, miR-132 was subsequently shown to regulate granulosa cell steroidogenesis of P4, discussed below. These miRNAs (miR-138 and miR-214), therefore, appear to suppress the bioavailability of cholesterol in granulosa cells, which is necessary for steroidogenesis (Figure 2). Downregulation by cAMP permits increased synthesis of steroid hormones like E2 during follicle growth.

Direct mechanisms for increased miRNA expression due to elevated cAMP, however, have not been thoroughly investigated. Deep RNA sequencing of mural and cumulus granulosa cells revealed miRNA located within the transcript for FSH and aromatase (33); intronic miRNA that are transcribed with their host gene have been shown to be abundant (40). The two miRNA identified, however, were previously unannotated and no follow-up has been conducted to further characterize their expression and function. Expression of miR-125b has been shown to be regulated by androgens in granulosa cells *via* extra- and intranuclear pathways (41). Coordinated kinase signaling regulates the ligand-bound androgen receptor nuclear localization where it binds to androgen response elements located in the miR-125b promoter to increase expression during follicle growth (41). This miRNA decreased apoptosis in both cultured granulosa cells and follicles *in vivo*, and was predicted to target the pro-apoptotic proteins Bcl-2 homologous antagonist/killer (BAK1), Bcl-2 modifying factor (BMF), Bcl-2-associated X protein (BAX), and tumor suppressor protein p53 (TP53), whose expression decreased following androgen administration (41), although no direct binding sites were tested. Availability of androgens during the follicular phase, therefore, has antiapoptotic effects on granulosa cells mediated at least in part by miRNA and is necessary for the downstream production of estrogens.

By far, the most common investigation centers around the miRNA regulation of E2 production by granulosa cells. We have investigated the role of miR-378 in regulating E2 production using pig granulosa cells as a model (42). Both mature miRNAs originating from the pre-miR-378 hairpin (designated miR-378-3p and -5p) are physiologically expressed in granulosa cells and were predicted to be conserved regulators of aromatase. Using a lentivirus expression vector to allow high efficiency delivery to primary cells, we transduced the recombinant lentivirus (miR-378) into granulosa cells to achieve over expression. While no significant change was detected at the mRNA level, aromatase protein and E2 levels were significantly decreased when miR-378 was exogenously expressed in granulosa cells derived from both small and large follicles, suggesting that the regulation of granulosa cell estradiol production by miR-378 occurs at the translational level. We confirmed the predicted binding sites for miR-378 in the aromatase 3′UTR using luciferase reporter assays in which mutation of the predicted miR-378 binding site abolished the observed reduction in luciferase activity (42). The inhibitory role of miR-378 on ovarian aromatase expression may also be indicative of an important physiological mechanism within the ovary that allows precise control over the levels of E2.

We have also extended our investigation to examine the role of miR-378 in cumulus granulosa cells and studied whether the manipulation of miRNA levels in cumulus cells could influence oocyte meiotic progression. Over expression of miR-378 decreased expansion of the cumulus oophorus and suppressed oocyte progression from the GV to MII stage by over 40% during *in vitro* oocyte maturation. Subsequent *in vitro* fertilization experiments confirmed an inhibitory effect of miR-378 on blastocyst formation (43). As we had previously seen, CYP19A1 expression was also decreased by miR-378 in cumulus cells, leading to a significant decrease in estradiol production (Table 1). Supplementing the maturation media with E2 could reverse the effects of miR-378 on cumulus expansion and meiotic progression (43), suggesting suppressed estradiol production *via* targeting aromatase may be one of the mechanisms by which miR-378 regulates the maturation of COCs.

**Table 1:** MicroRNAs that suppress estradiol release.

miRNA	Target gene(s)	Species	Tissue	Reference
51 miRNAs (miR-15a, miR-24, miR-25, miR-26a, miR-95, miR-96, miR-92, miR-108, miR-122, miR-124, miR-135, miR-144, miR-146, let-7d, let-7g, miR-1, miR-18, miR-19a, miR-20, miR-27a, miR-28, miR-29a, miR-98, miR-125a, miR-125b, miR-126, miR-137, miR-139, miR-148, miR-149, miR-184, miR-7, miR-10a, miR-22, miR-30a-3p, miR-31, miR-32, miR-34a, miR-101, miR-103, miR-105, miR-128, miR-129, miR-132, *miR-133A*, miR-140, miR-150, miR-151, miR-152, miR-187, miR-188)	Not identified	Human	Granulosa cells	(32)
miR-378	*CYP19A1*	Pig	Granulosa cells	(42)
miR-24	Not identified	Human	Granulosa cell line (KGN)	(44)
miR-320	*E2F1, SF-1*	Mouse, human	*In vivo* Injection of miR-320 (mouse)	(45)
miR-15a	Not identified	Human	Granulosa cells	(46)
miR-764-3p	*SF-1*	Mouse	Granulosa cells	(47)

Aside from directly targeting RNA messages for enzymes necessary for steroid hormone production, miRNA may also regulate E2 production indirectly *via* repression of transcription factors. The TGF-β1-regulated miRNA miR-764-3p suppressed the biosynthesis of E2 in mouse granulosa cells (47). No effect was seen on granulosa cell proliferation. Luciferase assays confirmed a binding site for miR-764-3p in the 3′UTR of steroidogenic factor (SF)-1, an important transcriptional activator for *CYP19A1* (47). Interestingly, SF-1 also acts as a transcriptional activator of Sarcoglycan zeta (*SGCZ*), the host gene for the intronic miR-383 (48). RNA binding motif, single stranded interacting protein (RBMS) 1, is a transcription factor that interacts with the master regulator c-Myc and is targeted by miR-383 (48). RBMS1 suppresses *CYP19A1* expression and thus E2 release. Together, this pathway was tested for its effects on E2 release from mouse ovarian granulosa cells. Loss and gain of function experiments tested each component in this pathway, and silencing of SF-1 attenuated miR-383-induced release of E2 (48). By regulating the transcription of aromatase these two miRNAs contribute to the regulation of E2 production in granulosa cells.

Fascinatingly, miR-133b was able to increase ovarian estradiol production in a similar manner by targeting the transcriptional repressor FOXL2 (Table 2). With two conserved sites located within its 3′UTR, FOXL2 was highly repressed when GCs were infected with adenovirus over expressing miR-133b (49). Chromatin immunoprecipitation using FOXL2 antibody showed decreased binding to both *STAR* and *CYP19A1* promoters with exogenous expression of miR-133b (49). Taken together, these studies begin to reveal a miRNA network regulating the expression of *CYP19A1* both through direct binding to mRNA messages and indirectly by targeting transcriptional regulators.

**Table 2:** MicroRNAs that increase estradiol release.

miRNA	Target gene(s)	Species	Tissue	Reference
miR-224	*SMAD4*	Mouse	Granulosa cells	(26)
miR-383	*RBMS1*	Mouse	Granulosa cells	(48)
miR-133b	*FOXL2*	Mouse, human	Granulosa cells	(49)
miR-132, miR-320, miR-520c-3p, miR-222	Not identified	Human	Granulosa cell line (KGN)	(44)
miR-132	*NURR1*	Mouse	Granulosa cells	(50)

The localization and functional impact in the ovary of the abundant miR-143 was also undertaken (51). This miRNA was highly expressed in granulosa cells where it appeared to regulate E2 production but not that of P4. Putative targets included 3β-HSD, CYP19A1, and 17β-HSD1. Unlike many studies of miRNA in the ovary, Zhang and colleagues attempted to elucidate the role for gonadotropes in regulating miR-143 expression. The lowest ovarian expression was seen during estrus, and isolated granulosa cells had lower miR-143 levels after treatment with FSH (51). Pretreatment of granulosa cells with small molecule inhibitors before FSH treatment pointed to the FSH-mediated PKA or PKC pathways as possible modes of action for miRNA expression. Further, LH served to increase miR-143 expression in the same cell type. Transfection of miR-143 mimics suppressed E2 levels in periovulatory granulosa cells. The only target identified for miR-143 in this study was KRAS proto-onco protein, which although relevant to cancer, has not been shown to be involved with FSH-mediated estradiol production (51). This report showed decreased E2 levels when cells were transfected with siRNA to KRAS, mimicking the effect of miR-143 over expression, but the magnitude of the change was substantially smaller than that of FSH or miR-143 mimics. This effect may have been due to proliferative effects on cultured granulosa cells.

### Luteal Phase

The feed forward loop between E2 and FSH drives changes in the follicle to prepare for ovulation. Follicle miR-320a was shown to be necessary for IGF-1-induced P4 and E2 production from cumulus cells. Cumulus cells taken from patients undergoing *in vitro* fertilization were treated with IGF-1 leading to increased levels of miR-320a, that was shown to directly target the osteogenic transcription factor RUNX2 (52). Promoter activity for both *CYP19A1* and *CYP11A1* suggested a direct role for RUNX2 in suppressing IGF-1-induced expression based on a luciferase reporter assay, although forced expression of RUNX2 had no suppressive effect on promoter activity without IGF-1 (52). Similar to the actions of miR-133b, miR-383, and miR-764-3p, hormone biosynthesis can be regulated indirectly by targeting transcription factors involved in their expression.

Regulation of the expression of LHR by miRNA has been the subject of numerous studies given its critical importance in ovulation. Based on microarray analysis of superovulated rat ovaries, LH was found to regulate the expression of miR-136-3p. Interestingly, this miRNA was also predicted, and ultimately shown to target the *LHCGR* message. Expression of this miRNA increased between 6 and 12 h after hCG administration, which corresponded to decreased *LHCGR* mRNA levels (53). A radioactive ^125^I-hCG assay was employed to detect surface expression of hCG-bound LHR. Exogenous miR-136-3p prevented the gradual increase in LHR at the surface seen with control vectors, while miR-136-3p inhibitor had the opposite effect (53). This suggests that miR-136-3p may play a role in the receptor internalization following ligand binding. In human granulosa cells, miR-513a-3p was shown to target three sites in the *LHCGR* mRNA, and mutation of at least two sites was necessary to increase relative luminescence in luciferase assays (54). Overexpression of this miRNA decreased LHCGR expression and significantly reduced hCG-stimulated cAMP levels in cultured human GCs when transfected with miR-513a (54).

A number of studies by the Menon group have also investigated the regulation of LHR by miRNA. Expression of miR-122 shows a transient spike after hCG administration *in vivo* and was suspected to play a role in the LHR down regulation observed after receptor activation (55). While no direct mechanism of action was tested, miR-122 was shown to be regulated by hCG *via* PKC and ERK1/2. Subsequent work by this group continued to investigate the PKC-miR-122-LHR axis and identified both LHR mRNA binding protein (LRBP) and SREBP as being up regulated by this miRNA, which leads to subsequent LHR degradation (56, 57). Further study is needed to understand how miR-122 exerts this effect.

An elegant study by Grossman demonstrated an important functional role for the LH-controlled miRNA miR-125-3p (58). Specifically, by targeting the Src family kinase FYN in granulosa cells, miR-125-3p attenuated cellular migration both *in vitro* and *in vivo*. Administration of hCG to PMSG-primed mice increased the expression of FYN while concomitantly decreasing miR-125-3p in granulosa cells. These cells displayed increased migration based on *in vitro* transwell assays, as would be expected during the extensive remodeling that occurs during the periovulatory stage. To confirm these findings *in vivo*, superovulated mice received intrabursal injections of either miR-125-3p mimics or FYN siRNA, both of which reduced the number of ovulated oocytes in mice (58). This study elucidates an important role of miRNA in mediating the granulosa cell response to LH. Rapid changes induced by the LH surge appear to be regulated epigenetically by miRNA networks in the somatic cells of the ovary to prepare for the release of a fertilizable oocyte.

Following ovulation, theca and granulosa cells continue to differentiate to a terminal luteal cell fate. Their steroidogenesis shifts toward P4 production. As with E2, the production of P4 and the regulation of its receptor are also regulated by miRNAs in ovarian somatic cells (Table 3). By over expressing miR-378 in cultured primary porcine granulosa cells, we revealed that miR-378-3p decreased protein levels and mRNA levels of PGR *via* targeting its 3′UTR. We observed that this regulation of PGR by miR-378-3p resulted in a corresponding decrease in gene transcripts of *ADAMTS1, CTSL1*, and *PPARG*, all known to be regulated by PGR and important for follicular maturation and remodeling (59). We speculate that decreasing miR-378 levels during the follicular phase allows granulosa cells to respond to increasing P4 concentrations and prepare for ovulation.

**Table 3:** Progesterone-related microRNAs.

miRNA	Target gene(s)	Species	Tissue	Impact	Reference
36 miRNAs (mir-15a, mir-24, mir-25, mir-26a, mir-95, mir-96, mir-92, mir-108, mir-122, mir-124, mir-135, mir-144, mir-146, let-7d, let-7g, mir-1, mir-18, mir-19a, mir-20, mir-27a, mir-28, mir-29a, mir-98, mir-125a, mir-125b, mir-126, mir-137, mir-139, mir-148, mir-149, mir-184, mir-7, mir-10a, mir-22, mir-30a-3p, mir-31, mir-32, mir-34a, mir-101, mir-103, mir-105, mir-128, mir-129, mir-132, *mir-133A*, mir-140, mir-150, mir-151, mir-152, mir-187, mir-188)	Not identified	Human	Granulosa cells	Decrease	(32)
10 miRNAs (miR-16, miR-24, miR-25, miR-122, miR-145, miR-182, miR-18, miR-125a, miR-147, miR-32, miR-103, miR-143, miR-150, miR-152, miR-153, miR-191)	Not identified	Human	Granulosa cells	Increase	(32)
miR-24, miR-193b, miR-483-5p	Not identified	Human	Granulosa cell line (KGN)	Decrease	(44)
miR-320	*E2F1, SF-1*	Mouse, human	*In vivo* injection of miR-320 (mouse)	Increase	(45)
miR-15a	Not identified	Human	Granulosa cells	Increase	(46)
miR-320a	*RUNX2*	Human	Cumulus cells	Increase	(51, 52)
miR-132	*STAR, MeCP2*	Rat	Granulosa cells		(60)

A study looking at the evolutionarily conserved miRNA family miR-132/212 found that the more prevalent member, miR-132, decreased bioavailable P4 in both adrenal and granulosa cells (60). Targeting two enzymes in the same steroidogenic pathway, this miRNA decreased the initial conversion of cholesterol to pregnenolone, a P4 precursor, and also suppressed MeCP2 expression, a negative regulator for 20α-HSD, which catalyzes the conversion of P4 into an inactive metabolite. The increase in miR-132 observed when granulosa cells were treated with a cAMP analog, suggests a possible regulation by FSHR or LHR, although a direct mechanism for expression was not investigated (60). Administration of miRNA inhibitors against miR-15a and miR-188 served to increase P4 release from cultured human granulosa cells, although no mechanism was elucidated (32). Although less well characterized than E2, it appears that a miRNA network also mediates the production and response to P4 in granulosa and luteal cells.

The CL is required during the earliest stages of pregnancy to maintain elevated progesterone levels. Studies in knockout mice confirmed that an ovary-specific lack of DICER, resulted in CL insufficiency and infertility in mice (61). This phenotype could be partially rescued, however, with an intrabursal injection of miR-17-5p and let-7b that were observed to regulate angiogenesis by targeting the tissue inhibitor or metalloproteinase 1 (TIMP1) (61). Not surprisingly, serum P4 levels failed to increase during pregnancy in mice with hypomorphic DICER expression in the CL (61). Despite ovulating normal, fertilizable oocytes, impaired angiogenesis negatively impacts the steroidogenic function of the CL and causes infertility. With the establishment of successful pregnancy, or a return to the estrous cycle, luteal cells undergo apoptosis and the CL begins to degenerate, in a process also known to be controlled be miRNA and the PGR (62, 63). While our understanding of the role played by miRNA during the establishment of pregnancy is still in its infancy, it appears likely that these small RNA have important functions in regulating this complex process.

## Conclusion and Perspectives

Hormone production and endocrine actions are important parts of ovarian function, with E2 and P4 the major steroid hormones generated in the ovary. In addition to their autocrine and paracrine function in ovarian follicular development and atresia, E2 and P4 also play important roles in mammary gland development, in establishing and maintaining uterine function, and in pregnancy. On top of the extensive list of regulators of E2 and P4 production and their receptor expression that have already been identified, the recent discoveries that they are also regulated by numerous miRNAs at different levels overlays another network controlling the synthesis and secretion of these important hormones.

We have begun to elucidate multiple roles for miRNA in the ovary with regards to integrating hormonal signals. While many facets of the E2 biosynthetic pathway have been examined, particularly aromatase both as a direct target of miRNA and indirectly *via* transcriptional repressors and activators, miRNA regulating other hormone pathways have been less well characterized. It is known that most mammalian mRNAs are targeted by multiple miRNAs, thus additional miRNAs that play a regulatory role in the expression of key enzymes involved in the biosynthesis of E2 and P4 may be identified in the near future. In addition, most of the data reported in this area come from *in vitro* studies of cultured granulosa cells. While these are important model systems for understanding the molecular mechanisms of miRNA regulation, these conclusions must be tested at the *in vivo* level. The well-documented spatial and temporal functions of miRNA suggest nuanced interactions that may be difficult to observe in isolated cells, particularly in a dynamic organ like the ovary. Recent advances in gene editing technology may be a promising means for further understanding the roles of miRNA in the regulation of ovarian steroidogenesis and the establishment of pregnancy *in vivo*.

Emerging data continue to point to new functional roles for miRNA. Direct actions of miRNA on gene expression have been suspected for some time, although insufficient data made this claim difficult to prove conclusively. It has recently been suggested, however, that miRNAs may bind to the promoter region of their target genes, and either activate or inhibit transcription. Additionally, miRNA may also be involved in the regulation of mRNA stability and alternative splicing in the nucleus [for review, see Ref. (64)]. While the majority of the work discussed above looked at canonical miRNA function (i.e., posttranslational repression of gene expression), future studies will reveal if similar non-canonical regulatory mechanisms exist in the ovary. If this is the case, the miRNA regulatory landscape of genes that are key to steroid hormone production will continue to expand.

Our understanding of ovarian miRNA continues to increase and new target genes are constantly being found. As work in this field progresses, new diagnostics and treatments for diseases such as polycystic ovarian syndrome (PCOS), the most prevalent endocrine disorder in young women, may be developed. Several studies have examined miRNA expression patterns in patients with PCOS (65–67). While miRNA profiling is not yet a routine diagnostic procedure, continued research in this area shows potential. Continued research into miRNA function in the ovary will broaden our understanding of fertility and ovarian function.

## Author Contributions

All the authors cowrote and edited the manuscript.

## Conflict of Interest Statement

The authors declare that the research was conducted in the absence of any commercial or financial relationships that could be construed as a potential conflict of interest.
